# Multiparametric MRI and artificial intelligence in predicting and monitoring treatment response in bladder cancer

**DOI:** 10.1186/s13244-024-01884-5

**Published:** 2025-01-02

**Authors:** Yuki Arita, Thomas C. Kwee, Oguz Akin, Keisuke Shigeta, Ramesh Paudyal, Christian Roest, Ryo Ueda, Alfonso Lema-Dopico, Sunny Nalavenkata, Lisa Ruby, Noam Nissan, Hiromi Edo, Soichiro Yoshida, Amita Shukla-Dave, Lawrence H. Schwartz

**Affiliations:** 1 Department of Radiology, Memorial Sloan Kettering Cancer Center, New York, NY USA; 2 Department of Radiology, Nuclear Medicine and Molecular Imaging, University Medical Center Groningen, Groningen, The Netherlands; 3 Dana-Farber Cancer Institute, Harvard Medical School, Boston, MA USA; 4 Department of Urology, Keio University School of Medicine, Shinjuku-ku, Tokyo Japan; 5 Department of Medical Physics, Memorial Sloan Kettering Cancer Center, New York, NY USA; 6 Office of Radiation Technology, Keio University Hospital, Shinjuku-ku, Tokyo Japan; 7 Department of Surgery, Urology Service, Memorial Sloan Kettering Cancer Center, New York, NY USA; 8 Department of Radiology, National Defense Medical College, Tokorozawa, Saitama Japan; 9 Department of Urology, Institute of Science Tokyo, Bunkyo-ku, Tokyo Japan

**Keywords:** Artificial intelligence, Biomarker, Multiparametric magnetic resonance imaging, Treatment response, Urinary bladder neoplasm

## Abstract

**Abstract:**

Bladder cancer is the 10th most common and 13th most deadly cancer worldwide, with urothelial carcinomas being the most common type. Distinguishing between non-muscle-invasive bladder cancer (NMIBC) and muscle-invasive bladder cancer (MIBC) is essential due to significant differences in management and prognosis. MRI may play an important diagnostic role in this setting. The Vesical Imaging Reporting and Data System (VI-RADS), a multiparametric MRI (mpMRI)-based consensus reporting platform, allows for standardized preoperative muscle invasion assessment in BCa with proven diagnostic accuracy. However, post-treatment assessment using VI-RADS is challenging because of anatomical changes, especially in the interpretation of the muscle layer.

MRI techniques that provide tumor tissue physiological information, including diffusion-weighted (DW)- and dynamic contrast-enhanced (DCE)-MRI, combined with derived quantitative imaging biomarkers (QIBs), may potentially overcome the limitations of BCa evaluation when predominantly focusing on anatomic changes at MRI, particularly in the therapy response setting. Delta-radiomics, which encompasses the assessment of changes (Δ) in image features extracted from mpMRI data, has the potential to monitor treatment response. In comparison to the current Response Evaluation Criteria in Solid Tumors (RECIST), QIBs and mpMRI-based radiomics, in combination with artificial intelligence (AI)-based image analysis, may potentially allow for earlier identification of therapy-induced tumor changes.

This review provides an update on the potential of QIBs and mpMRI-based radiomics and discusses the future applications of AI in BCa management, particularly in assessing treatment response.

**Critical relevance statement:**

Incorporating mpMRI-based quantitative imaging biomarkers, radiomics, and artificial intelligence into bladder cancer management has the potential to enhance treatment response assessment and prognosis prediction.

**Key Points:**

- Quantitative imaging biomarkers (QIBs) from mpMRI and radiomics can outperform RECIST for bladder cancer treatments.
- AI improves mpMRI segmentation and enhances radiomics feature extraction effectively.
- Predictive models integrate imaging biomarkers and clinical data using AI tools.
- Multicenter studies with strict criteria validate radiomics and QIBs clinically.
- Consistent mpMRI and AI applications need reliable validation in clinical practice.

**Graphical Abstract:**

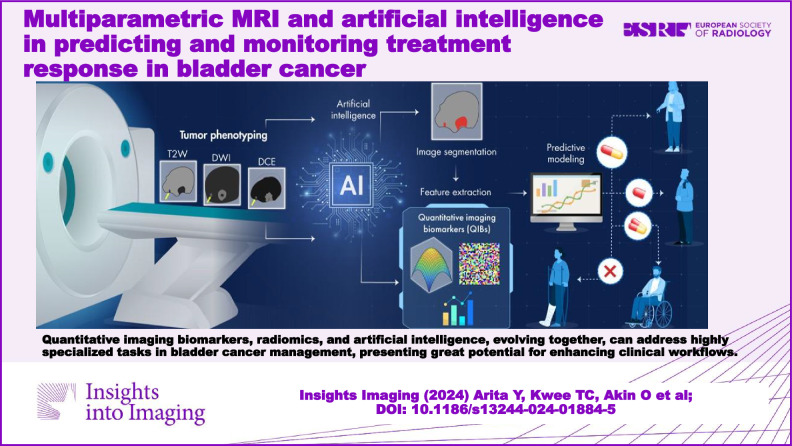

## Introduction

Bladder cancer (BCa) is the 10th most common and 13th most deadly cancer worldwide. Its incidence is steadily rising, especially in Europe. Urothelial carcinomas account for over 90% of BCa cases, while the remaining 10% comprise less common histologies such as squamous cell carcinoma and adenocarcinomas [1]. The invasion of the muscularis propria significantly affects BCa management and prognosis, with muscle-invasive BCa (MIBC) having a worse prognosis than non-muscle-invasive BCa (NMIBC) [2, 3]. Distinguishing between these two types is crucial [4]. NMIBC is typically managed with transurethral resection of bladder tumor (TURB), with or without intravesical therapy [2, 3]. MIBC is usually treated with radical cystectomy (RC), often combined with neoadjuvant systemic therapy, or with a multimodal bladder-preservation approach involving TURB, radiation therapy (RT), and chemotherapy [2, 3].

MRI provides proper local assessment and monitoring of treatment response. The Vesical Imaging Reporting and Data System (VI-RADS), a consensus multiparametric magnetic resonance imaging (mpMRI)-based reporting system, has been proposed to standardize pre-operative muscle invasion assessment in BCa patients [4, 5].

This review aims to provide an update on the potential utility of imaging modalities in BCa, explore their expanded role in treatment response assessment, and discuss the emerging importance of artificial intelligence (AI) in this setting. Therefore, the first half of this review discusses the significance and challenges of MRI in prognostication and assessment of treatment response, and the second half describes the application of AI to mpMRI and radiomics to overcome these challenges.

## Current diagnostic and treatment strategies in bladder cancer patients

**Fig. 1:**
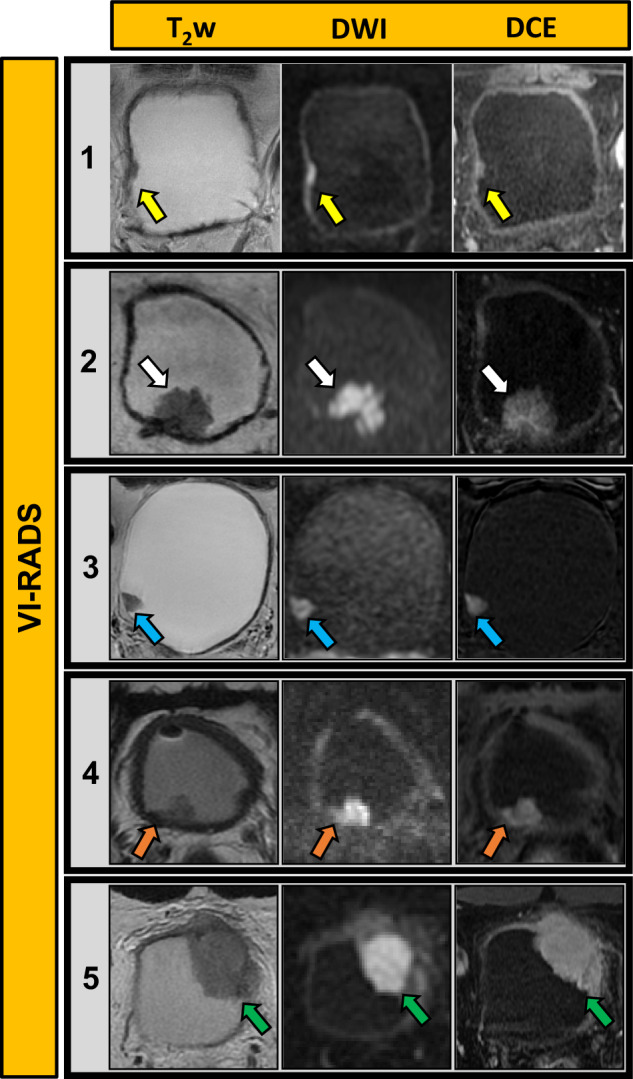
VI-RADS scoring in representative bladder cancer patients. VI-RADS 1: Lesion size < 1 cm; VI-RADS 2: Exophytic tumor with stalk or sessile/broad-based tumor with thickened inner layer; VI-RADS 3: No category 2 findings, but no clear disruption of muscularis propria; VI-RADS 4: Extension of the tumor tissue into the muscularis propria; and VI-RADS 5; Extension of the tumor tissue into the extravesical fat. BCa, bladder cancer; DCE, dynamic contrast-enhanced; DW, diffusion-weighted; T2w, T2-weighted imaging; VI-RADS, Vesical Imaging Reporting and Data System

**Fig. 2:**
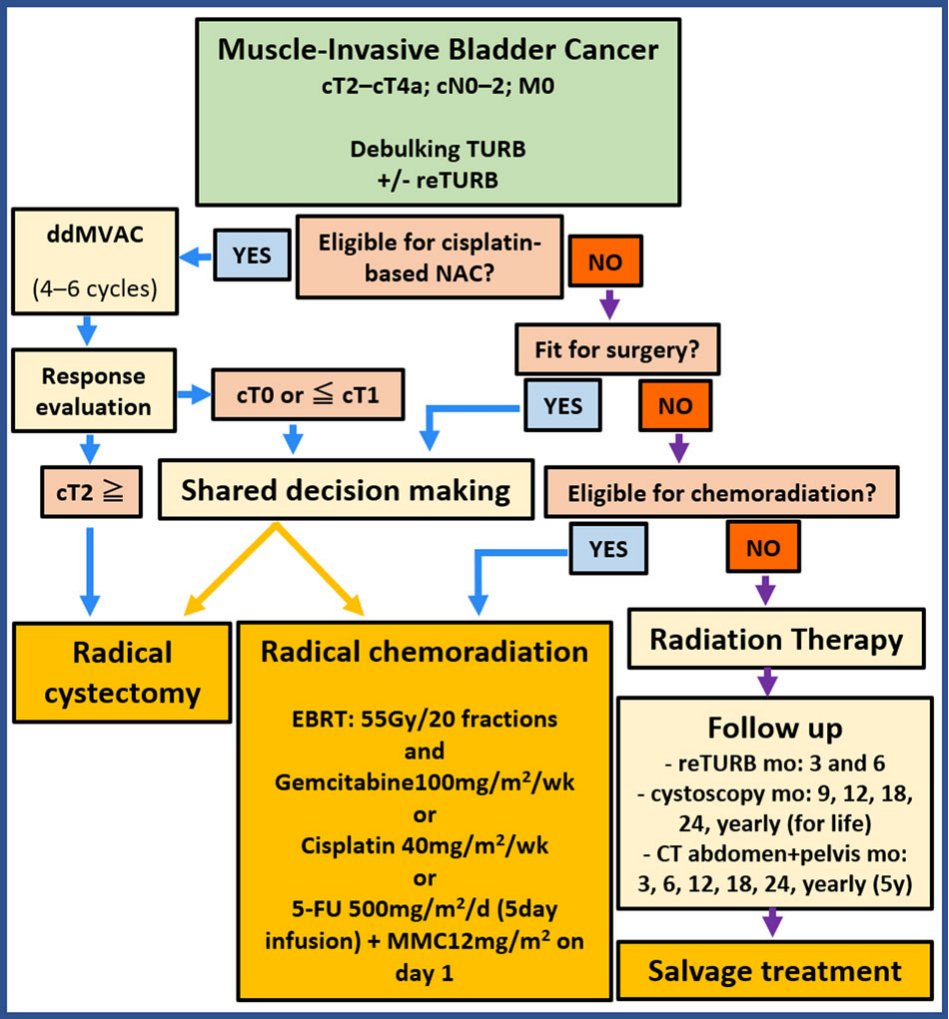
Flow chart of the current treatment planning process in MIBC patients. 5-FU, 5-fluorouracil; ddMVAC, dose-dense methotrexate, vinblastine, adriamycin, and cisplatin; EBRT, external beam radiation therapy; MIBC, muscle-invasive bladder cancer; MMC, mitomycin C; NAC, neoadjuvant chemotherapy; TURB, transurethral resection of bladder tumor

The initial diagnostic workup for BCa, which commonly presents with hematuria, typically involves physical examination, ultrasonography (US), and diagnostic cystoscopy [2, 6]. The integration of mpMRI into BCa guidelines is progressing [2]. While CT urography is used for initial imaging evaluation, MRI provides better local assessment and monitoring of treatment response. Several studies confirmed the diagnostic accuracy of VI-RADS [7–11]. However, applying VI-RADS to post-treatment bladder wall assessment is challenging due to anatomical changes, particularly in the muscle layer. Figure 1 illustrates VI-RADS scoring in representative patients. Table 1, Supplementary Tables 1 and 2 detail the international guidelines, TNM classification, and characteristics of conventional imaging modalities, respectively [2, 4–6, 12–21]. For NMIBC, the 2021 European Association of Urology scoring model aids in risk stratification and guiding adjuvant treatments such as Bacillus Calmette-Guérin (BCG) therapy or chemotherapy [22–25]. Immunotherapies such as Pembrolizumab are emerging as second-line treatment in BCG-unresponsive NMIBC [26, 27]. In MIBC, RC remains the standard treatment, often integrated with neoadjuvant chemotherapy (NAC), because of MIBC’s heterogeneity in response to conventional regimens [28, 29]. Recent advancements have made RC viable for elderly patients, although many succumb within a year post-surgery, highlighting the need for alternative therapies to reduce mortality and preserve the quality of life [30–33]. Bladder-sparing multimodal therapy, involving comprehensive TURB followed by NAC and chemoradiation therapy (CRT) presents a promising curative alternative to RC [2, 34]. Figure 2 depicts the decision-making process for managing MIBC patients.

**Table 1:** Summary of international guidelines for recommended diagnostic approaches in bladder cancer patients

European Association of Urology (EAU) [2]	
Patient situation	Recommendations
Bladder tumor detection	Direct to rigid cystoscopy and TURB High sensitivity of US, CT, and MRI in detecting bladder tumors
Confirmed MIBC via biopsy	Chest, abdomen, and pelvic CT imaging for staging CT urography for urinary tract assessment

American Urological Association (AUA) [89]	
Risk level of BCa	Recommendations
Low	Shared decision-making with physicians, follow-up urinalysis after 6 months
Intermediate	Renal ultrasonography, optical cystoscopy evaluation
High	CT urography, cystoscopy Post-cystoscopy: CT/MRI of pelvis and abdomen (with/without contrast) before TURB

National Institute for Health and Care Excellence (NICE) [90]	
Risk level of BCa and Patient situation	Recommendations
Bladder tumor staging	CT or MRI with excretory phase and chest CT
High risk of metastatic disease, e.g., locally advanced primary tumors, visible regional adenopathy	FDG-PET-CT imaging

*BCa* bladder cancer, *FDG* fluorodeoxyglucose, *MIBC* muscle-invasive bladder cancer, *PET* positron emission tomography, *TURB* transurethral resection of bladder tumor, *US* ultrasonography

## Next-generation imaging techniques for treatment assessment in bladder cancer patients

### QIBs derived from mpMRI

Diffusion-weighted (DW)- and dynamic contrast-enhanced (DCE)-MRI are part of the mpMRI VI-RADS protocol [5], and quantitative imaging biomarkers (QIBs) derived from these methods can help evaluate early cellular and vascular changes at baseline and in response to treatment [35, 36]. In addition, physiological QIBs can reveal spatial and temporal alterations in cancer cells and the tumor microenvironment before observable size changes [37, 38].

mpMRI is well-suited for radiomics, which involves the mathematical extraction of data (such as signal intensities and pixel- or voxel-based relationships) from medical images [39–42]. Changes in radiomic values between pre- and post-treatment (delta-radiomics) may enhance the current standard for monitoring therapeutic response, Response Evaluation Criteria in Solid Tumors (RECIST), by identifying early tumor changes [39, 40, 43].

QIBs from DW- and DCE-MRI hold promise in predicting early treatment response as non-invasive alternatives to cystoscopy [35, 36, 44].

The apparent diffusion coefficient (ADC) map, which reflects the mobility of water molecules in tissues, is calculated from DW images with at least two *b*-values [45]. Water diffusion is thought to be inversely correlated with tumor tissue cellularity [46]. The advanced diffusion kurtosis imaging (DKI) model extends diffusion-derived parameters beyond the ADC to quantify the deviation of water diffusion from a Gaussian distribution. The kurtosis coefficient (*K*) is a dimensionless quantity reported to differ between healthy and tumor tissue [47].

**Fig. 3:**
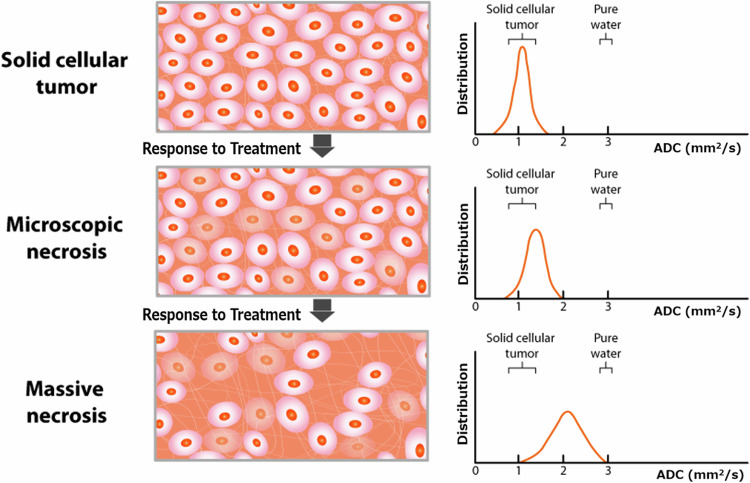
Mechanism of ADC from DW-MRI in response to treatment. The ADC map, representing the average movement of water molecules in tissues, is generated from DW-MRI using at least two *b*-values. Free water diffusion is restricted by barriers such as intact cell membranes, which are inversely related to the cellularity of tumor tissue. Reference: [37] ADC, apparent diffusion coefficient; DW, diffusion-weighted

**Fig. 4:**
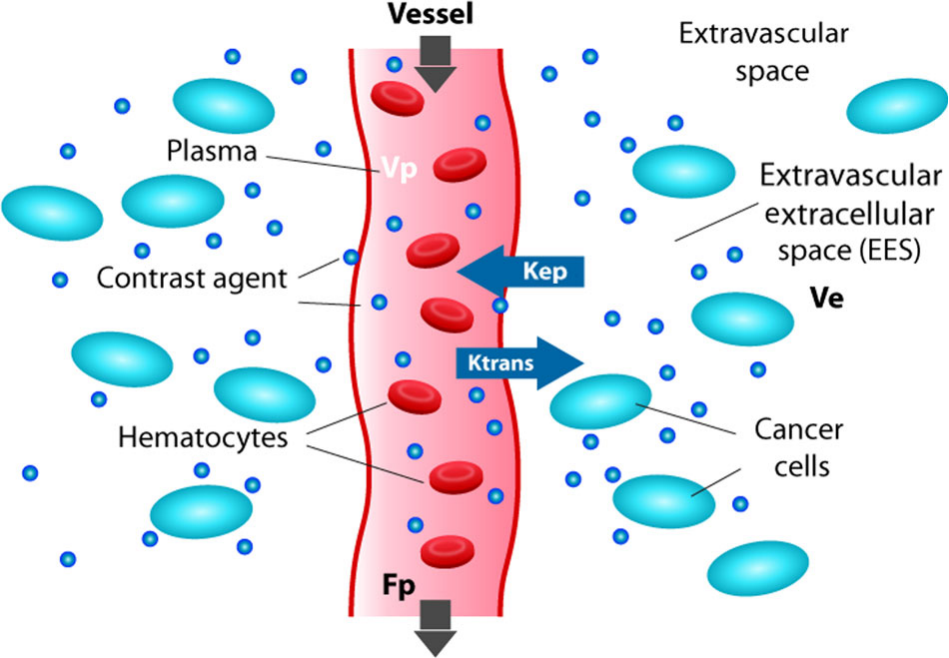
Mechanism of DCE-MRI. The signal intensity time course of DCE-MRI data can be modeled using both semiquantitative and quantitative techniques. Semiquantitative parameters include signal enhancement, wash-in, and wash-out rates. Quantitative analysis involves modeling the time-course data of the contrast agent concentration curve to estimate the volume transfer constant, which indicates vascular perfusion and permeability. Reference: [37] DCE, dynamic-contrast-enhanced; EES,  extravascular extracellular space; Fp, flow blood plasma; *K*_ep_, rate constant of contrast agent from EES to blood plasma; *K*^*trans*^, volume transfer constant from blood plasma to EES; Ve, EES volume; Vp, plasma blood volume

DCE-MRI is a potentially useful method to provide physiological QIBs. Using semiquantitative and quantitative approaches, the time course of signal intensity of DCE-MRI data can be modeled [48]. The semiquantitative parameters include signal enhancement, wash-in, and wash-out rate. Quantitative analysis of time course-contrast agent concentration curves data to estimate the volume transfer constant and reflect vascular perfusion/permeability [48]. Integration of QIBs into clinical protocols could potentially reduce the need for cystoscopy and enable a less invasive determination of treatment response assessment. Figures 3 and 4 show the mechanisms of ADC from DW-MRI and the quantitative parameters from DCE-MRI, respectively.

### Radiomics and AI

**Fig. 5:**
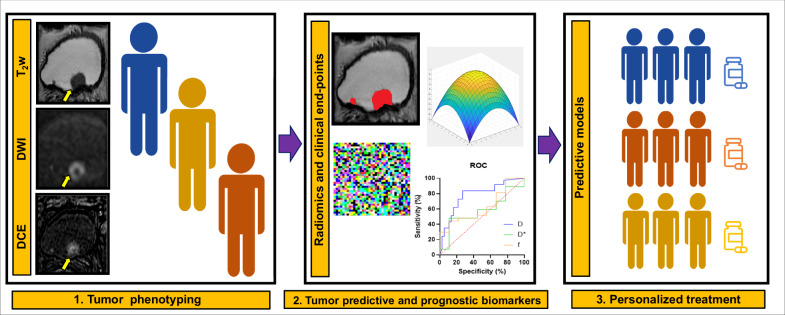
QIBs and Radiomics that may be used to apply personalized care in bladder cancer patients. DCE, dynamic contrast-enhanced; DW, diffusion-weighted; QIB, quantitative imaging biomarker; T2w, T2-weighted imaging

Radiomics involves the computerized extraction of multiple quantitative imaging features, including information on size and shape and advanced pixel- or voxel-based texture features. Changes in radiomics-derived values between pre- and post-treatment (delta-radiomics) may enhance the current standard for monitoring therapeutic responses by identifying early tumor changes [6, 16, 19]. Over the past decade, significant advancements in preoperative imaging methods have been achieved, notably with mpMRI of the bladder, which has become a cornerstone in diagnosing and staging BCa, offering up to 85% accuracy in detecting muscle invasion [49]. The emergence of radiomics, which quantifies the heterogeneity of medical images through feature extraction that is not visible to the naked eye, such as pixel or voxel intensity and texture derivatives, has marked a potential leap in radio-oncology [39, 50, 51]. Figure 5 illustrates a scheme for QIBs and radiomics, which may be used to apply personalized care in patients with BCa.

## Pretreatment prognosis prediction

Prognostic prediction of BCa outcomes following therapies such as BCG, NAC, and multimodality therapy using pretreatment MRI-based QIBs, and radiomics is a promising area of research.

Table 2 summarizes the literature on QIBs from mpMRI and radiomics for prognosis prediction using pretreatment MRI.

**Table 2:** Quantitative imaging biomarkers from multiparametric MRI and Radiomics for predicting prognosis in patients with bladder cancer using pretreatment MRI

Therapy	Reference	Number of patients	Metrics	Reported cut-off values and performances	Inference	Strengths	Limitations
BCG	Abd Elwahab KM, et al [53]	65	ADC	For the recurrence prediction, ADC < 1.09 × 10^−3^ mm^2^/s For the progression prediction, ADC < 0.985 × 10^−3^ mm^2^/s	Lower ADC tumors in pre-BCG MRI exhibited significantly higher recurrence and progression rates	ADC value, in conjunction with other risk stratifications, will have a promising role in stratifying patients with T1G3 who need to proceed to early radical cystectomy versus conservative treatment	Relatively small sample size. Longer periods of follow-up are needed for the prognosis data collection.
NAC	Nguyen HT, et al [54]	20	ADC histograms	N/A	Uniformity and entropy metrics derived from ADC histogram in pre-NAC MRI were significantly higher in responders than non-responders	Voxel-wise ADC values can quantify and characterize the microcellular heterogeneity in bladder tumors that may be useful in the prediction of response to NAC before treatment to ensure optimal outcomes and improve the patient’s quality of life	A relatively small number of cases and the survival data in this study are still preliminary
	Zhang X, et al [55]	58	ADC histograms	For the NAC responder diagnosis, Mean ADC: 1.33 × 10^−3^ mm^2^/s, with an AUC of 0.88.	ADC histogram parameters (mean and percentiles) in pre-NAC MRI were significantly higher in responders than non-responders	Both pretreatment ADC values measured from single-section ROI and pretreatment ADC histogram parameters obtained from the whole-tumor VOI were able to predict NAC response for MIBC	The sample size was relatively small. For patients with multiple lesions, only the largest lesion was selected
	Hafeez S, et al [56]	48	ADC histograms	ΔADC > 15.5% at the 75th percentile was associated with significant improvement in OS, CSS, PFS and time to cystectomy	75th percentile ADC from ADC histogram in pre-NAC MRI was significantly correlated with progression-free survival	Quantitative ADC analysis can successfully identify NAC response and improve long-term clinical outcomes	Relatively small sample size.
	Zhang X, et al [57]	112	Radiomics derived from T_2_w: LoG-sigma-5-0-mm-3D-NGTDM; wavelet-LHH-GLDM Radiomics derived from ADC: wavelet-HLL-GLRLM- run entropy; wavelet-LLL-first order 10 percentile; wavelet-LLH-GLCM-Difference Average	For the responder prediction, The combined radiomics model based on T2WI, DWI, and ADC yielded the highest AUC of 0.967.	Radiomics features, such as wavelet-transformed features in pre-NAC MRI were significantly associated with the response to NAC	A significant difference was found in cT stage between the two groups. Lesions with the cT2 stage accounted for a larger proportion than lesions with the cT3 stage in the responders’ group, while a larger proportion of lesions with the cT3 stage was found in the non-responders’ group. The Model T2WI achieved the best predictive performance with the highest AUC among the single-modality radiomics models	Due to the small sample size, no model validation using a separate cohort was performed
Chemoradiotherapy	Kimura K, et al [58]	45	Radiomics derived from ADC: First quartile (Q1 ADC): GLCM; GLCM homogeneity	For the pCR prediction, The SVM model derived from texture analysis of ADC map provided with the highest AUC of 0.96	Radiomics features, such as lower Q1 ADC and GLCM homogeneity in pre-NAC MRI, were significantly associated with the response to CRT	ADC map–based texture analysis could be a new approach for predicting the CRT response of MIBC. The addition of a second-order TF to the Q1 ADC value of the conventional index improved the predictive performance of CRT sensitivity; the SVM model incorporating Q1 ADC, GLCM correlation, and GLCM homogeneity was more accurate in predicting CRT sensitivity than the mean ADC value	The study was a single-center retrospective with a relatively small cohort size

*AUC* area under the curve, *BCG* Bacillus Calmette-Guérin, *CR* complete response, *CRT* chemoradiotherapy, *CSS* cancer-specific survival, *DW* diffusion-weighted, *GLCM* gray-level co-occurrence matrix, *GLRLM* gray-level run-length matrix, *mpMRI* multiparametric MR, *NAC* neoadjuvant chemotherapy, *NGTDM* neighboring gray-tone difference matrix, *OS* overall survival, *pCR* pathological complete response, *PFS* progression-free survival, *SVM* support vector machine, *T2WI* T2-weighted imaging

Note: The table cites the major publications in this area

## Prediction by mpMRI

BCG therapy, primarily used for NMIBC, involves the introduction of a weakened strain of tuberculosis bacteria into the bladder to stimulate the immune system and target cancer cells [52]. Routine cystoscopy and urinalysis are essential for local monitoring, because BCG -induced inflammation can impair the diagnostic performance of standard imaging techniques. In T1 high-grade BCa patients treated with BCG, Abd Elwahab et al reported lower ADC values on pretreatment MRI were associated with a significantly higher risk of disease recurrence and progression than higher ADC values [53]. Specifically, multivariate analysis showed that ADC < 1.09 × 10^-3 ^mm²/s and ADC < 0.985 × 10^-3 ^mm²/s were the only significant independent predictor for recurrence and predictor for progression, respectively [53]. These findings suggest that ADC values could help categorize patients with T1 high-grade BCa who might require early RC instead of conservative treatment with BCG.

**Fig. 6:**
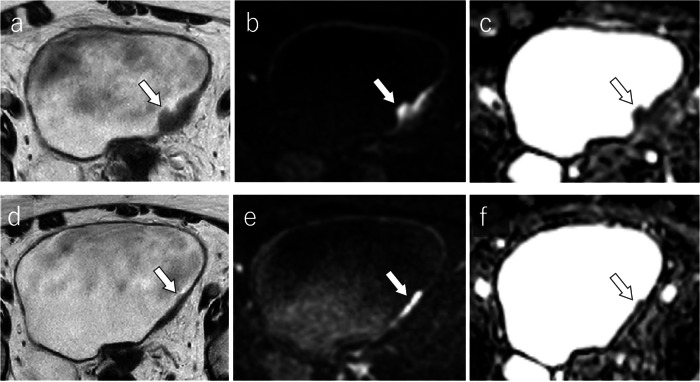
NAC responder: a 76-year-old woman with a 24-mm urothelial carcinoma located in the left posterior bladder wall pre-NAC MRI shows a sessile and broad-based tumor with localized wall thickening and lack of clear disruption of the muscularis layer on T2WI (**a**) [T2WI VI-RADS score: 3], high-signal intensity on DW-MRI (**b**) [DW VI-RADS score: 3], and homogeneous low signal intensity on the ADC map with a mean ADC value of 0.579 × 10^−3^ mm^2^/s (**c**). The tumor is indicated by a white arrow. Post-NAC MRI demonstates reduced tumor volume with slight wall thickening on T2WI (**d**), linear high signal intensity on DW-MRI (**e**), and linear low signal intensity on ADC map with a mean ADC value of 0.982 × 10^−3^ mm^2^/s (**f**). The tumor is indicated by a white arrow. ADC, apparent diffusion coefficient; DW, diffusion-weighted; NAC, neoadjuvant chemotherapy; T2WI, T2-weighted imaging; VI-RADS, vesical imaging-reporting data system

ADC values on pretreatment MRI provide a valuable prognostic tool for T1 high-grade BCa patients undergoing BCG therapy, potentially guiding treatment decisions towards more aggressive interventions when necessary. Figure 6 shows a representative case of BCG therapy.

Nguyen et al revealed that uniformity and entropy metrics derived from ADC histograms on pretreatment DW-MRI were significantly higher in responders than in non-responders [54]. Zhang et al reported that mean ADC values in pretreatment MRI were significantly higher in responders than non-responders, using the reported cut-off value of 1.33 × 10^-3 ^mm^2^/s with an AUC of 0.88 for the responder diagnosis [55]. Hafeez et al reported that the changes between baseline ADC and post-radiotherapy ADC (ΔADC) > 15.5% at the 75th percentile were associated with significant improvement in overall survival (hazard ratio (HR), 0.40; 95% confidence interval (CI), 0.19–0.86; *p* = 0.0179), BCa-specific survival (HR, 0.26; 95% CI, 0.08–0.82; *p* = 0.0214), progression-free survival (HR, 0.16; 95% CI, 0.05–0.48; *p* = 0.0012), and time to cystectomy (HR, 0.19; 95% CI, 0.07–0.47; *p* = 0.0004) [56].

ADC histogram metrics from pretreatment DW-MRI hold promise as prognostic indicators for NAC response, aiding in the stratification of patients likely to benefit from treatment.

## Prediction by radiomics

Zhang et al reported high accuracy in predicting tumor response to NAC using a model that combined T2-weighted imaging (T2WI), DW-MRI, and ADC features, particularly wavelet-transformed features, with an AUC of 0.967 [57]. Radiomics features such as wavelet-HLL-GLRLM (Gray Level Run Length Matrix) run entropy and wavelet-LLH-GLCM (Gray Level Co-occurrence Matrix) difference average in pretreatment MRI were significantly associated with response to NAC [57].

The integration of wavelet-transformed features from T2WI, DW-MRI, and ADC maps significantly enhances the accuracy of predicting NAC responses, underscoring the potential of radiomics in personalized treatment planning.

Multimodality therapy, combining TURB with CRT, offers an organ-sparing alternative to RC. Response rates and overall survival outcomes vary, and ongoing research aims to identify predictive factors for treatment response to optimize patient selection [2, 34]. Kimura et al used radiomics to predict CRT response using imaging features such as Q1 ADC, GLCM correlation, and GLCM homogeneity [58]. Lower Q1 ADC and GLCM homogeneity on pre-treatment MRI significantly correlated with CRT responsiveness. The developed support vector machine model derived from texture analysis of the ADC map provided the highest AUC of 0.96 for predicting pCR. The GLCM correlation assesses the linear dependence of the grayscale values of pixel pairs, whereas the GLCM homogeneity measures the uniformity of the distribution of elements in the GLCM matrix. Elevated homogeneity values imply a consistent texture that is useful for differentiating normal from pathological tissues, as cancerous formations typically show reduced homogeneity [58].

**Fig. 7:**
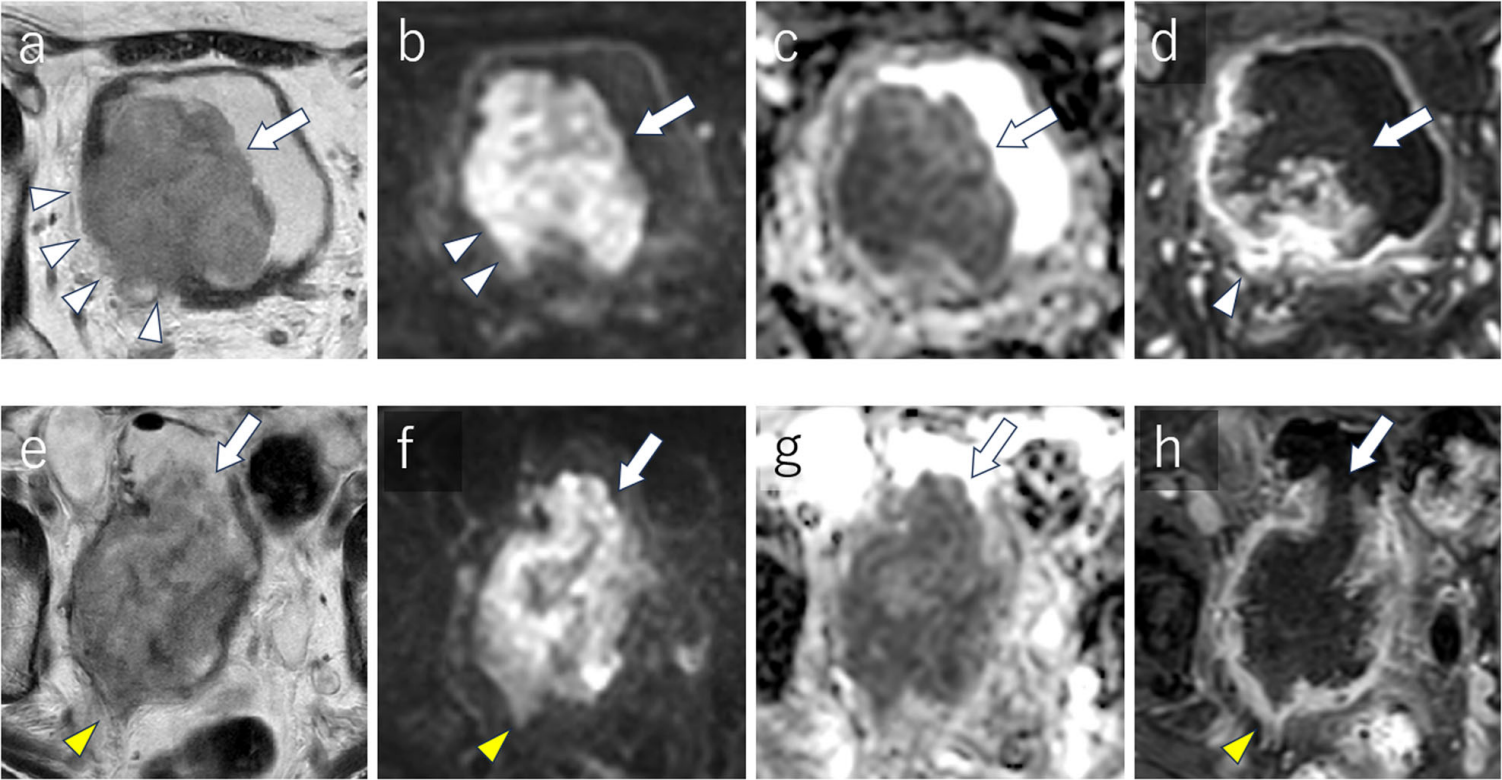
NAC non-responder: a 71-year-old man with a 65-mm urothelial carcinoma with squamous differentiation the right posterior bladder wall Pre-NAC MRI reveals a lobulated tumor with partial disruption in the muscle layer on T2WI (**a**) [T2WI VI-RADS score: 4], high-signal intensity on DW-MRI indicating infiltration into the muscle propia (**b**) [DW VI-RADS score: 4], heterogeneous low signal intensity on the ADC map with a mean ADC value of 0.994 × 10^−3^ mm^2^/s (**c**), and DCE-MRI shows heterogeneous enhancement and muscle layer disruption (**d**) [DCE VI-RADS score: 4]. After one course of gemcitabine and cisplatin, and two courses of gemcitabine and docetaxel due to renal dysfunction, post-NAC MRI shows tumor expansion and invasion into the perivesical fat on T2WI (**e**) [T2WI VI-RADS: 5] and DW-MRI (**f**) [DW VI-RADS: 5], with a corresponding low signal intensity on the ADC map (a mean ADC value: 0.906 × 10^−3^ mm^2^/s) (**g**). DCE-MRI confirms perivesical infiltration (**h**) [DCE VI-RADS: 5]. The tumor is indicate white arrows, muscle infiltration by white arrowheads, and perivesical fat infiltration by yellow arrowheads. ADC, apparent diffusion coefficient; DCE, dynamic contrast-enhanced imaging; DW, diffusion-weighted neoadjuvant chemotherapy; T2WI, T2-weighted imaging; VI-RADS, vesical imaging-reporting and data system

Multimodality therapy as a bladder-sparing treatment is an emerging therapeutic strategy, and further application of radiomics and AI in this field, along with validation in multicenter studies, is a promising area of research. Figure 7 shows a representative case of a multimodal therapy responder.

## Treatment response assessment after NAC

### Characterization by mpMRI

Patients achieving pathological complete response (pCR) at post-NAC exhibit better outcomes, whereas those with residual disease face a higher risk of recurrence and poorer prognoses [2, 59, 60]. Early and accurate assessment of NAC response can help to replace ineffective treatments, improve outcomes, and reduce side effects [59, 60]. Ahmed et al reported that ADC became significantly higher and more heterogeneous in responders due to decreased microcellularity after NAC. They reported that using a cutoff value of the mean ADC in post-NAC MRI of 0.911 × 10^-3^ mm²/s, the sensitivity and specificity for diagnosing pCR were 90.0% to 91.2% and 89.3% to 91%, respectively [61]. DW-MRI can help identify good responders to NAC in MIBC and optimize patient selection for bladder-sparing approaches. Hafeez et al observed significant differences in mean ADC changes between responders and non-responders, using a reported cut-off value after NAC of 1.65 ×10^-3^ mm²/s for mean ADC with an AUC of 0.74 for the responder diagnosis [56]. Donaldson et al found that combining quantitative and semi-quantitative DCE-MRI analyses could effectively differentiate residual tumors from treated areas without residual tumors. The sensitivity and specificity for differentiating residual tumors from treatment effect were 70% and 100% for rSI80s (relative enhancement in the venous phase), 60% and 86% for Fp (plasma perfusion), and 75% and 100% for both metrics combined [48]. Chakiba et al showed that rSI80s measured in residual tumor areas were significantly higher than those in treated areas without residual tumor, using the rSI80s at 40% with an AUC of 0.861, indicating the potential of rSI80s to assess NAC response [62]. Nguyen et al demonstrated significant associations between changes in volume fractions (VFs) and the NAC response [63]. Responsive tumors showed an increase in VF of cluster 2 (low *k*_ep_ (rate constant of contrast agent efflux) and high Amp (amplitude or initial area under the concentration curve)) and a reduction in clusters 1 (low *k*_ep_ and low Amp) and 3 (high *k*_ep_ and low Amp) from baseline to mid-cycle. The VF change of cluster 2 presented with sensitivity/specificity/accuracy of 96%/100%/97%, respectively [63]. Wang et al reported higher mean kurtosis (MK) values in residual tumors compared to inflammatory lesions [64]. Reported cutoff mean kurtosis values were MKa = 0.637, MKb = 0.516, and MKc = 0.478, respectively. The MKb presented with the highest AUC of 0.943 [64].

The nacVI-RADS is a novel iteration of the VI-RADS score that has recently been studied in the post-treatment context and includes specific categories tailored for assessing the response to NAC [65]. The nacVI-RADS categories have been reported to correspond with the findings from the final RC pathology in cases of pCR and patients exhibiting partial or minimal response to NAC [65]. Further investigations using a large-scale cohort are warranted to validate this scoring system.

**Fig. 8:**
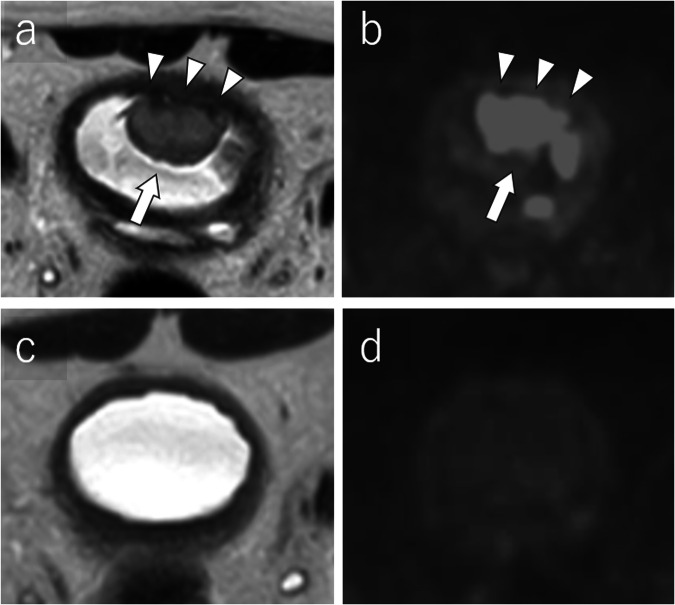
Immunotherapy responder: a 76-year-old man with a 35-mm urothelial carcinoma in the anterior bladder wall pre-immunotherapy MRI shows a round-shaped tumor with no apparent muscularis disruption on T2WI (**a**) [T2WI VIRADS score: 3] and homogeneous high signal on DW-MRI (**b**) [DW VI-RADS score: 3]. Post-immunotherapy MRI reveals significant tumor reduction, with no apparent lesions on T2WI (**c**) or DW-MRI (**d**). The tumor is indicated by white arrow, and intact muscle layers by white arrowheads. ADC, apparent diffusion coefficient; DCE, dynamic contrast-enhanced; DW, diffusion-weighted; T2WI, T2-weighted imaging; VI-RADS, vesical imaging-reporting and data system

**Fig. 9:**
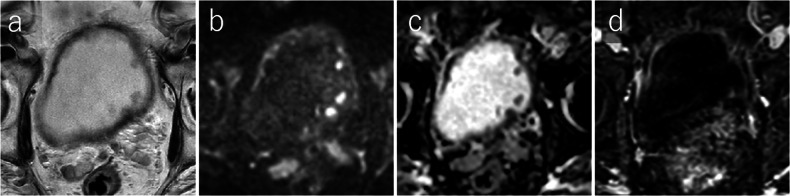
Post-BCG therapy: an 81-year-old man with multiple papillary nodules and broad wall thickening post-BCG therapy MRI shows intermediate signal intensity papillary nodules with broad wall thickening on T2WI (**a**), high signal intensity on DW-MRI (**b**), intermediate signal intensity on the ADC map with a mean ADC value of 1.272 × 10^−3^ mm^2^/s (**c**), and DCE-MRI shows multiple contrast-enhanced nodules (**d**). ADC, apparent diffusion coefficient; DW, diffusion-weighted; T2WI, T2-weighted imaging; VI-RADS, vesical imaging-reporting and data system

Although the integration of mpMRI and specific biomarkers, such as ADC and DCE-MRI parameters, show promise for the noninvasive assessment of NAC response, standardization of the measurement techniques and validation through large-scale studies are necessary to enable widespread clinical application. Figures 8 and 9 show representative cases of NAC responders and non-responders, respectively.

## Posttreatment prognosis prediction

An accurate assessment of treatment response is crucial for improving the prognosis of patients with BCa. However, the standard RECIST criteria based on tumor size are limited in their ability to accurately evaluate treatment response [51]. Table 3 summarizes the literature on QIBs from mpMRI and radiomics to evaluate treatment response using post-treatment MRI.

**Table 3:** Quantitative imaging biomarkers from multiparametric MRI and Radiomics for characterizing treatment response in patients with bladder cancer using post-treatment MRI

Therapy	Reference	Number of patients	Metrics	Reported cut-off values and performances	Inference	Strengths	Limitations
NAC	Ahmed SA, et al [61]	90	ADC and wash-out rate	For the pCR diagnosis, Mean ADC: 0.911 × 10^−3^ mm^2^/s, with AUCs of 0.911 to 0.921. Wash-out rate: 0.677 min^−1^, with AUCs of 0.880 to o.895.	ADC in post-NAC MRI became significantly higher and more heterogeneous in responders than non-responders due to the lower micro-cellularity after NAC pCR is negatively correlated with a wash-out rate in post-NAC MRI	DW-MRI could identify good responders to NAC in MIBC and may help optimize patient selection for bladder-sparing approaches. The wash-out rate also could effectively identify MIBC patients with pCR after NAC, and combined DW-MRI and DCE-MRI helped identify pCR	Need for standardization of ADC measurement technique
	Hafeez S, et al [56]	48	ADC	For the NAC responder diagnosis, Post NAC mean ADC: 1.65 × 10^−3^ mm^2^/s, with AUC of 0.74.	ΔADC (mean and percentiles) significantly increased in responders than in non-responders in post-NAC MRI	DW-MRI-derived metrics could be non-invasive biomarkers for NAC assessment and sensitivity	NAC response was not defined pathologically in some of the enrolled patients
	Donaldson SB, et al [48]	21	rSI_80s_ Fp	For the residual tumor diagnosis, rSI_80s:_ 2.6, with sensitivity/specificity of 70%/100% Fp: 19.0, with sensitivity/specificity of 60%/86%	rSI_80s_ and Fp measured in areas of residual tumor were significantly higher than those measured in treated areas without residual tumor in post-NAC MRI	rSI_80s_ and F_p_ had the highest sensitivity and specificity of the parameters estimated for differentiation between areas of residual tumor and NAC effect; the sensitivity and specificity rose when the two parameters were combined	The variation in the number of days between the completion of chemotherapy and the second DCE-MRI examination may have led to slightly less homogeneous groups for statistical comparison post-treatment in this relatively small number of patients
	Chakiba C, et al [62]	12	rSI_80s_	For the residual tumor diagnosis, rSI_80s_: 40%, with an AUC of 0.861	rSI_80s_ measured in areas of residual tumor were significantly higher than those measured in areas of treatment effect in post-NAC MRI	Performing pre- and early post-treatment DCE-MR imaging could be useful to obtain an early evaluation of tumor response to NAC for localized muscle-invasive. Significant pre- and post-treatment decrease in tumor size and thickness was also found for patients with complete pathological response	The study used a semi-quantitative approach reporting the variation of SI
	Nguyen HT, et al [63]	30	VFs	For the NAC responder diagnosis, ΔVF for three clusters (cluster 1: low *k*_ep_ and low Amp, cluster 2: low *k*_ep_, and cluster 3: high Amp, high *k*_ep_, and low Amp) is 4%, 1%, and –1%. ΔVF of cluster 2 presented with the highest AUC of 0.96.	The changes of VFs in post-NAC MRI are significantly associated with response to NAC due to the spatial heterogeneity of chemotherapeutic response within a bladder tumor	Responsive bladder tumors showed an increase in the VF of cluster 2 (low *k*_ep_ and high Amp) and a reduction in the VFs of both clusters 1 (low *k*_ep_ and low Amp) and 3 (high *k*_ep_ and low Amp) from baseline to mid-cycle. Non-responders had an increase in the VFs of clusters 1 and 3 and a decrease in the VF of cluster 2	The number of non-responders was relatively small (*n* = 7)
	Wang F, et al [64]	50	DKI	For the residual tumor diagnosis, Mean kurtosis: MKa: 0.637, MKb: 0.516, and MKc: 0.478. The MKb presented with the highest AUC of 0.943.	MK (MKa, MKb, MKc) from DKI in post-NAC MRI was significantly higher in residual bladder tumors than in inflammatory lesions	Kurtosis could be a more reliable method than conventional DW-MRI to discriminate malignant from benign tissue in the post-TUR/chemotherapy state	The sample size was relatively small
Immunotherapy	Necchi A, et al [73]	82	mpMRI including DW-MRI	In post-pembrolizumab MIBC, the interobserver variability of mpMRI assessment: *κ*-values ranging from 0.5 to 0.76.	Proposed stepwise assessment incorporating post-immunotherapy mpMRI sequence	In post-pembrolizumab MIBC, mpMRI assessment had acceptable interobserver variability and represented the basis for a radiological CR/NED status definition predicting the pCR to pembrolizumab	These results may not necessarily translate into a survival benefit in an immunotherapy setting
RT	Hafeez S, et al [75]	34	ADC	For the radiotherapy response diagnosis, ΔADC mean: 0.16 × 10^−3^ mm^2^/s, with an AUC of 0.97.	Mean ADC became significantly higher in responders than non-responders in post-RT MRI due to the lower micro-cellularity derived from the tumor cell death, edema, inflammation, and microvessel leakage	DW-MRI-derived biomarkers could provide a non-invasive tool for bladder cancer radiotherapy response assessment and aid risk stratification	Only half of the patient cohort had evaluable tumors

*CR* complete response, *DCE* dynamic contrast-enhanced, *DKI* diffusion kurtosis imaging, *DW* diffusion-weighted, *Fp* plasma perfusion, *MK* Median kurtosis, *mpMRI* multiparametric MRI, *NAC* neoadjuvant chemotherapy, *NED* no evidence of disease, *pCR* pathological complete response, *rSI80s* relative enhancement at the venous phase, *RT* radiation therapy, *VFs* volume fractions

Note: The table cites the major publications in this area

## Characterization with mpMRI

### Immunotherapy

Immunotherapy is the cornerstone of cancer treatment. Traditionally, the RECIST criteria monitor solid tumor responses via imaging, particularly CT scans [66–68]. However, unique response patterns to immunotherapy, such as pseudo-progression [69, 70], challenge the RECIST framework, leading to adaptations, such as the Immune-Related Response Criteria and modified RECIST [71, 72]. Necchi et al evaluated the bladder tumor response following neoadjuvant pembrolizumab treatment and proposed a stepwise assessment incorporating mpMRI sequences. In post-pembrolizumab MIBC, mpMRI assessment had acceptable inter-observer variability (*κ*-values ranging from 0.5 to 0.76) and forms the basis for defining radiological complete response status/no evidence of disease, predicting pCR to pembrolizumab [73].

**Fig. 10:**
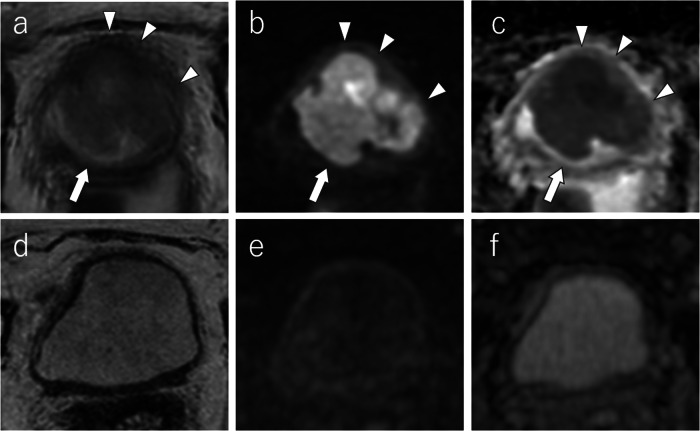
Trimodality therapy responder: a 74-year-old woman with a 53-mm urothelial carcinoma in the anterior bladder wall Pre-therapy MRI shows a lobulated tumor with partial disruption of the muscle layer on T2WI (**a**) [T2WI VI-RADS score: 4], high signal intensity on DW-MRI suggesting muscle infiltration (**b**) [DW VI-RADS score: 4], and homogeneous low signal intensity on the ADC map with a mean ADC value of 1.160 × 10^−3^ mm^2^/s (**c**). Post-therapy MRI, after trimodality therapy consisting of transurethral resection of bladder tumor (TURB), two courses of cisplatin treatment, and intensity-modulated radiation therapy (IMRT 60 Gy/30 fr), demonstrates significant tumor reduction with no apparent lesions on T2WI (**d**), DW-MRI (**e**), or the ADC map (**f**). The tumor is indicated by a white arrow, and disruptions or infiltrations by white arrowhead. ADC, apparent diffusion coefficient; DW-MRI, diffusion-weighted; IMRT, intensity-modulated radiation therapy T2-weighted imaging; TURB, transurethral resection of the bladder; VI-RADS, vesical imaging-reporting and data system

While early findings indicated that mpMRI can be valuable in assessing immunotherapy response, further research is essential to confirm its efficacy and potential survival benefits. The development of standardized protocols is crucial for broader clinical applications. Figure 10 shows a representative case of an immunotherapeutic responder.

### Radiation therapy (RT)

Radiation therapy strategies aim to optimize disease control while minimizing damage to the healthy bladder tissues [74]. Hafeez et al reported that post-radiation ADC was significantly higher in responders owing to lower microcellularity from tumor cell death, edema, inflammation, and microvessel leakage [75]. The changes between baseline ADC mean and post-radiotherapy ADC mean (ΔADC mean) of 0.16 × 10^-3^ mm^2^/s diagnosed radiotherapy response with sensitivity/specificity/positive predictive value/negative predictive value of 92.9%/100.0%/100.0%/75.0%, respectively. DW-MRI-derived biomarkers could provide a noninvasive tool for BCa radiotherapy response assessment and aid in risk stratification [75].

The use of mpMRI, particularly DW-MRI, in evaluating RT response is promising, but further validation is required.

## Characterization with radiomics

No established papers on radiomics have been published for treatment response assessment utilizing post-treatment MRI. The absence of established radiomics research in this setting highlights a significant opportunity for future studies.

## Application of AI into mpMRI and radiomics

### Segmentation and feature extraction

**Fig. 11:**
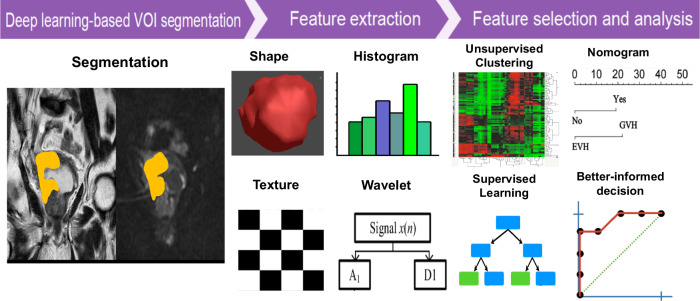
Deep learning-based segmentation and radiomics feature extraction. Deep learning-based segmentation is a technique using artificial intelligence models to automatically identify and delineate regions of interest within medical images, enhancing precision and efficiency in medical imaging analysis. Subsequent radiomics feature extraction is the process of converting medical images into high-dimensional data by extracting a large number of quantitative features. These features can be used for predicting disease outcomes, characterizing tumors, and aiding in personalized treatment planning. VOI, volume of interest

Within bladder MRI, AI-based segmentation algorithms, especially those predicated on deep learning (DL), have played a pivotal role in the standardization of radiomics feature extraction. For example, CNNs can be trained on a dataset of bladder mpMRI scans to autonomously segment relevant regions and quantify pertinent features such as tumor texture, edge definition, and contrast variations, which are traditionally labor-intensive tasks subject to observer variability. This automation facilitates large-scale radiomic analyses and promotes uniformity in feature extraction across different studies, thereby diminishing the variability introduced by manual segmentation or feature computation [76]. Moribata et al have recently demonstrated that an MRI-based U-net model exhibits high precision in segmenting BCa on mpMRI, with the radiomic features extracted showing notable reproducibility. The multi-sequence imaging model achieved the highest Dice Similarity Coefficient through five-fold cross-validation, with mean Dice Similarity Coefficient values of 0.83 and 0.79 for the training and validation datasets, respectively [77]. Figure 11 shows a flowchart of DL-based segmentation and feature extraction.

### Accelerating image acquisition

**Fig. 12:**
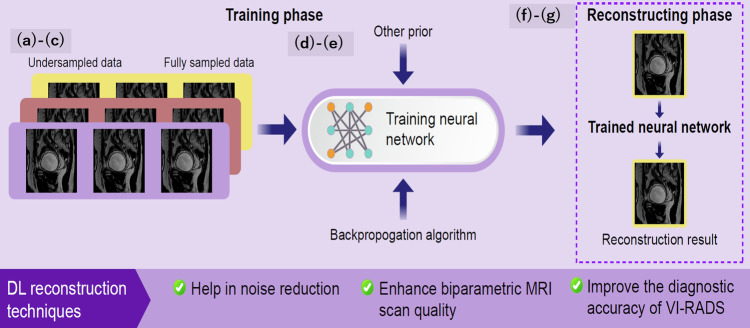
Mechanism of deep learning-based MRI reconstruction. Deep learning-based MRI reconstruction uses neural networks to improve the quality and speed of MR image reconstruction. **a** Data acquisition: Raw MR data, known as *k*-space data, is acquired using MR scanners. The *k*-space data represents the spatial frequency information of the scanned object. **b** Data Preprocessing: The acquired k-space data is preprocessed to normalize and prepare it for the neural network. This may include steps like zero-filling, undersampling, and noise reduction. **c** Neural Network Architecture: A deep learning model, typically a convolutional neural network or a variant like U-Net, is designed to handle the reconstruction task. The network is trained on paired datasets of undersampled k-space data and their corresponding fully sampled ground-truth images. **d** Training Phase: The model learns to map undersampled k-space data to high-quality reconstructed images. It minimizes the difference between the predicted images and the ground-truth images using a loss function, often involving mean squared error or other metrics relevant to image quality. **e** Inference Phase: The trained model is used to reconstruct new MR images from undersampled *k*-space data. The model processes the input data to produce high-quality, fully reconstructed images. **f** Postprocessing: The reconstructed images may undergo additional postprocessing steps to enhance visual quality and ensure clinical usability. This can include filtering, edge enhancement, and artifact correction. **g** Output: The final output is a high-quality MR image reconstructed faster and potentially with fewer artifacts than traditional methods. DL, deep learning; VI-RADS, Vesical Imaging Reporting and Data System

DL reconstruction techniques have shown the potential to reduce scan durations and enhance noise reduction in bladder MRI protocols. A recent study indicated that integrating T2WI with denoising DL reconstruction significantly enhances the diagnostic accuracy of VI-RADS for muscle invasion in patients with BCa compared to conventional T2WI scans [78]. Furthermore, the application of denoising DL reconstruction on biparametric MRI (a combination of T2WI and DW-MRI without DCE-MRI) has improved the diagnostic accuracy for muscle invasion over conventional biparametric MRI [79]. This DL reconstruction method could be particularly advantageous for patients with BCa requiring frequent monitoring to evaluate tumor response to treatment. Figure 12 illustrates the mechanism of DL-based MRI reconstruction.

### Minimizing contrast agent dosages or eliminate the need for contrast agents

Advanced AI techniques, such as generative adversarial networks, hold the potential to revolutionize contrast-enhanced bladder MRI by reducing the necessary dosage of contrast agents or eliminating their requirement entirely. Generative adversarial networks have been investigated for their capability to augment non-contrast images or simulate contrast enhancement, delivering essential diagnostic information while reducing the risks associated with gadolinium-based agents [80, 81]. Although this innovative technique has not been previously applied to bladder MRI protocols, further pioneering studies are warranted to demonstrate how generative adversarial networks could be utilized in bladder MRI to generate diagnostically relevant images from non-contrast or low-dose contrast scans, suggesting a move towards safer imaging practices for patients with renal impairment due to systemic chemotherapy.

## Challenges for QIBs and radiomics

### Challenges for QIBs from mpMRI

Despite promising applications of QIBs from mpMRI in BCa treatment response assessment, their integration into routine clinical practice still faces significant hurdles. The Quantitative Imaging Biomarkers Alliance (QIBA) highlights these challenges. It provides recommendations on reducing the variability in MR protocols and inconsistencies in data processing techniques, which impact the precision and standardization of QIB measurements, such as ADC from DW-MRI and *K*^*trans*^ from DCE-MRI [35]. A lack of repeatability and reproducibility of data impedes clinical translation, necessitating standardized imaging protocols and robust data analysis methods to improve measurement reliability [35, 82]. QIBA emphasizes the need for stringent quality control measures and recommends the use of standardized phantoms to ensure consistent calibration and performance of MR systems. Extensive test-retest studies are crucial for establishing the precision and accuracy of QIBs, involving both phantom studies and clinical trials with standardized protocols. Additionally, robust statistical methods are needed to assess technical performance, including the determination of repeatability and within-subject coefficient of variation to evaluate measurement variability [35, 83]. Addressing these challenges through standardized protocols, rigorous quality control, and extensive validation studies are essential for integrating the QIBs. It is also important to successfully translate QIBs into routine clinical practice to improve the BCa treatment response assessment.

### Challenges for radiomics

Despite the promising applications of radiomics in BCa treatment response assessment, challenges remain, particularly in harmonizing imaging data owing to the variability in MR protocols and acquisition parameters across different vendors and devices, impacting the consistency of radiomic features [84, 85]. Traditional and more advanced image harmonization methods, including Gaussian mixture-based ComBat normalization, have been proposed to address these issues, although they still depend on predefined groupings and feature sets [86]. Additionally, the reliability of radiomics feature extraction depends on the chosen region of interest, with variations between and within readers potentially impacting the extracted features and decisions based on them. Robustness analysis tackles this by examining how minor image perturbations affect features. Features that remain stable despite these perturbations are preferred, leading to more reliable diagnoses [87]. While most BCa radiomics features have previously demonstrated good-to-excellent reproducibility, further assessment is needed to determine their generalizability to external data and the effect of stability thresholds on test performance [77].

### METhodological RadiomICs Score (METRICS)

One of the main challenges in radiomics research is the need for reproducibility stemming from inadequate study designs and methodological descriptions. Kocak et al introduced the METhodological RadiomICs Score (METRICS), a comprehensive quality scoring tool for radiomics research endorsed by the European Society of Medical Imaging Informatics (EuSoMII), to address this issue [88]. Table 4 summarizes the contents of the METRICS tool. This tool was developed by a large international panel of experts using a modified Delphi protocol that involved three stages and four rounds of consensus building to ensure robustness and broad acceptance. METRICS aims to assess and improve the methodological quality of radiomics studies by addressing the translational gap between radiomics research and clinical practice.

**Table 4:** A summary of the METRICS tool

Categories	Contents no.	Evaluation criteria	Weights (%)	Score^f^
Study design	#1	Adherence to radiomics and/or machine learning-specific checklists or guidelines	3.68	
	#2	Eligibility criteria that describe a representative study population	7.35	
	#3	High-quality reference standard with a clear definition	9.19	
Imaging data	#4	Multicenter	4.38	
	#5	Clinical translatability of the imaging data source for radiomics analysis	2.92	
	#6	Imaging protocol with acquisition parameters	4.38	
	#7	The interval between imaging used and reference standard	2.92	
Segmentation^a^	#8	Transparent description of segmentation methodology	3.37	
	#9	Formal evaluation of fully automated segmentation^b^	2.25	
	#10	Test set segmentation masks produced by a single reader or automated tool	1.12	
Image processing and feature extraction	#11	Appropriate use of image preprocessing techniques with transparent description	6.22	
	#12	Use of standardized feature extraction software^c^	3.11	
	#13	Transparent reporting of feature extraction parameters, otherwise providing a default configuration statement	4.15	
Feature processing	#14	Removal of non-robust features^d^	2.00	
	#15	Removal of redundant features^d^	2.00	
	#16	Appropriateness of dimensionality compared to data-sized	3.00	
	#17	Robustness assessment of end-to-end deep learning pipelines^e^	2.00	
Preparation for modeling	#18	Proper data partitioning process	5.99	
	#19	Handling of confounding factors	3.00	
Metrics and comparison	#20	Use of appropriate performance evaluation metrics for task	3.52	
	#21	Consideration of uncertainty	2.34	
	#22	Calibration assessment	1.76	
	#23	Use of uni-parametric imaging or proof of its inferiority	1.17	
	#24	Comparison with a non-radiomic approach or proof of added clinical value	2.93	
	#25	Comparison with simple or classical statistical models	1.76	
Testing	#26	Internal testing	3.75	
	#27	External testing	7.49	
Open science	#28	Data availability	0.75	
	#29	Code availability	0.75	
	#30	Model availability	0.75	
Total METRICS score (should be given as a percentage)				
Quality category^g^				

Reference: [88] ^a^ Conditional for studies including region/volume of interest labeling

^b^ Conditional for studies using fully automated segmentation

^c^ Conditional for hand-crafted radiomics

^d^ Conditional for tabular data use

^e^ Conditional on the use of end-to-end deep learning

^f^ Score is simply the weight if present and 0 otherwise

^g^ Proposed total score categories: 0 ≤ score < 20%, “very low”; 20 ≤ score < 40%, “low”; 40 ≤ score < 60%, “moderate”; 60 ≤ score < 80%, “good”; and 80 ≤ score ≤ 100%, “excellent” quality

The METRICS tool encompasses 30 items organized into nine categories: study design, imaging data, segmentation, image processing and feature extraction, feature processing, preparation for modeling, metrics and comparison, testing, and open science. Each category addresses critical aspects of radiomics research methodologies. The items were carefully selected through a systematic literature review and expert consensus to ensure that they covered essential methodological considerations [88]. For instance, the study design category emphasizes adherence to radiomics-specific checklists or guidelines, defining eligibility criteria, and using high-quality reference standards. Imaging data items address multicenter studies, clinical translatability, and imaging protocols. Segmentation items focus on transparent methodology and the evaluation of automated segmentation. Image processing and feature extraction include appropriate preprocessing techniques and transparent reporting [88].

A significant innovation of METRICS is the weighting of categories and items based on expert opinions, using a hierarchical ranking method. This approach ensured that the most critical aspects of radiomics research had a greater influence on the final score. For instance, study design and proper data partitioning have higher weights than open science practices, which, while important, were considered secondary in the context of study design robustness.

## Conclusions

Although challenges remain for QIBs from mpMRI and radiomics in surpassing current standards based on RECIST in real-world clinical practice, they hold the potential for assessing early response and guiding therapy, particularly for newer BCa treatments. AI may enhance this process by accurately segmenting and analyzing mpMRI, improving the extraction of radiomics features, and aiding the development of predictive models that combine imaging biomarkers with clinical data. According to the EuSoMII and QIBA guidelines, validating the results of Radiomics and QIBs through large-scale multicenter studies and dedicated validation criteria is essential to ensure the consistency and reliability of mpMRI and AI applications in clinical practice.

## Supplementary information

ELECTRONIC SUPPLEMENTARY MATERIAL

## Data Availability

The data that support the findings of this study are available from the corresponding author, upon reasonable request.

## Abbreviations

AI: Artificial intelligence
BCa: Bladder cancer
BCG: Bacillus Calmette-Guérin
CI: Confidence interval
CRT: Chemoradiotherapy
DCE: Dynamic contrast-enhanced
DKI: Diffusion kurtosis imaging
DL: Deep learning
DW: Diffusion-weighted
EuSoMII: European Society of Medical Imaging Informatics
GLCM: Gray-level co-occurrence matrix
HR: Hazard ratio
*K* or *K*_app_: Kurtosis coefficient
METRICS: METhodological RadiomICs Score
MIBC: Muscle-invasive bladder cancer
mpMRI: Multiparametric MRI
NAC: Neoadjuvant chemotherapy
NMIBC: Non-muscle-invasive bladder cancer
pCR: Pathological complete response
QIB: Quantitative imaging biomarker
QIBA: Quantitative imaging biomarkers alliance
RC: Radical cystectomy
RECIST: Response Evaluation Criteria in Solid Tumors
RT: Radiation therapy
T2WI: T2-weighted imaging
TURB: Transurethral resection of bladder tumor
VI-RADS: Vesical Imaging Reporting and Data System
