# Supplementary material for: Multiparametric MRI and artificial intelligence in predicting and monitoring treatment response in bladder cancer
Source: Insights Imaging. 2025 Jan 2;16:7. doi: 10.1186/s13244-024-01884-5 (PMC11695553; doi:10.1186/s13244-024-01884-5)
Supplement: Supplementary file 1 — ELECTRONIC SUPPLEMENTARY MATERIAL [file 13244_2024_1884_MOESM1_ESM.pdf]

Multiparametric MRI and Artificial Intelligence in Predicting and Monitoring Treatment Response in Bladder Cancer
ELECTRONIC SUPPLEMENTARY MATERIAL

Supplementary Table 1: TNM classification

TNM Classification of Bladder Cancer	
**Primary Tumor (T)**	
**Tx**	Tumor cannot be evaluated
**T0**	Tumor is not evident
**Ta**	Noninvasive papillary carcinoma
**Tis**	Flat tumor: carcinoma is in situ
**T1**	Tumor invades mucosa and submucosa
**T2**<br><br>**T2a**<br>**T2b**	Tumor invades perivesical tissue<br><br>superficial muscle<br>deep muscle
**T3**<br><br>**T3a**<br>**T3b**	Tumor invades perivesical tissue<br><br>microscopically<br>macroscopically
**T4**<br><br>**T4a**<br>**T4b**	Tumor invades adjacent organs<br><br>prostate, seminal vesicles, uterus, or vagina<br>pelvic and abdominal wall
**Lymph Nodes (N)**	
**Nx**	Lymph nodes cannot be evaluated
**N0**	No evidence of regional lymphadenopathies
**N1**	Metastasis in a single lymph node (hypogastric, obturator, external iliac, presacral)
**N2**	Metastasis in multiple lymph nodes (hypogastric, obturator, external iliac, presacral)
**N3**	Metastasis in common iliac lymph nodes
**Distant Metastasis (M)**	
**M0**	No evidence of distant metastasis
**M1**<br><br>**M1a**<br>**M1b**	Metastasis<br><br>metastasis in non-regional lymph nodes<br>other distant metastases

Supplementary Table 2: Characteristics of conventional imaging modalities in patients with bladder cancer

Imaging Modality	Characteristics and Key Points
**US Imaging [11-13]**	The efficacy of contrast-enhanced and three-dimensional US methods is currently being explored. Given its moderate sensitivity in identifying lower and upper urinary tract alterations, the US may supplement physical examination. It can identify focal thickening of the bladder wall, renal masses, and hydronephrosis. However, the US cannot ascertain the specific cause of hematuria, and because a negative US result does not rule out the presence of upper tract urothelial carcinoma, follow-up with CT urography is required. Consequently, the US is insufficient as the sole imaging modality for diagnosing hematuria.
**CT Urography [11-14]**	CT urography with intravenous contrast is essential for diagnosis of suspected BCa. This modality comprehensively examines the urinary tract and can incorporate an excretory phase (10-16 minutes post-contrast injection) alongside corticomedullary and nephrographic phases. It can identify papillary tumors, typically manifest as filling defects, and detect hydronephrosis. Comparison of CT urography vs. cystoscopic evaluation for BCa detection showed an overall sensitivity of 79% vs. 95%, specificity of 94% vs 92%, accuracy of 91% vs. 93%, PPV of 75% vs. 72%, and negative NPV of 95% vs. 99%. CT urography exhibits an elevated NPV of 98% in patients assessed solely for hematuria. The evidence suggests that CT urography holds value in detection, but more advanced imaging techniques are required to address staging and risk assessment.
**MRI [2; 4; 5; 15-20]**	mpMRI is emerging as the optimal imaging modality for regional staging of BCa due to its superior soft tissue contrast resolution, lack of ionizing radiation, and heightened sensitivity in delineating tumor infiltration of the bladder wall and the extent of perivesical fat or adjacent organ invasion. This approach includes acquiring multiplanar images with high spatial and contrast resolution, encompassing morphological T2WI, DCE- and DW- MRI sequences, with the ADC map derived from DW-MRI. The primary utility of mpMRI lies in local tumor staging, but it can also monitor therapeutic response and detect potential local disease recurrence.<br>Challenges associated with implementing mpMRI protocols in the radiological workflow led to the development of the VI-RADS by an international consensus of imaging experts in BCa. The VI-RADS scoring system facilitates the standardization of bladder MRI acquisition protocols, interpretation, and reporting, paralleling the RADS framework.
**FDG-PET-CT [21]**	FDG-PET-CT's utility for local disease staging is limited, mainly due to urinary excretion. Alternative isotopes and

	receptor-specific molecules are being investigated to expand their potential. Further studies are needed to evaluate the impacts of radiotherapy, endoscopic intervention, and intravesical chemotherapy on FDG-PET interpretation within the bladder. Imaging sensitivity with 18-FDG PET may decrease 50% following chemotherapy, necessitating cautious interpretation of results following systemic treatment.

BCa, Bladder cancer; DCE, Dynamic contrast-enhanced; DW, Diffusion-weighted; FDG, Fluorodeoxyglucose; mpMRI, Multiparametric MRI; NPV, Negative predictive value; PPV, Positive predictive value; T2WI, T2-weighted imaging; VI-RADS, Vesical Imaging Reporting and Data System.
